# The compensatory phenomenon of the functional connectome related to pathological biomarkers in individuals with subjective cognitive decline

**DOI:** 10.1186/s40035-020-00201-6

**Published:** 2020-05-27

**Authors:** Haifeng Chen, Xiaoning Sheng, Caimei Luo, Ruomeng Qin, Qing Ye, Hui Zhao, Yun Xu, Feng Bai, for the Alzheimer’s Disease Neuroimaging Initiative

**Affiliations:** 1 Department of Neurology, Drum Tower Hospital, Medical School and The State Key Laboratory of Pharmaceutical Biotechnology, Institute of Brain Science, Nanjing University, 321 Zhongshan Road, Nanjing, Jiangsu 210008 P. R. China; 2 Jiangsu Key Laboratory of Molecular Medicine, Medical School of Nanjing University, Nanjing, China; 3 Jiangsu Province Stroke Center for Diagnosis and Therapy, Nanjing, China; 4 Nanjing Neuropsychiatry Clinic Medical Center, Nanjing, China

**Keywords:** Subjective cognitive decline, rs-fMRI, Machine learning, Compensatory mechanism

## Abstract

**Background:**

Subjective cognitive decline (SCD) is a preclinical stage along the Alzheimer’s disease (AD) continuum. However, little is known about the aberrant patterns of connectivity and topological alterations of the brain functional connectome and their diagnostic value in SCD.

**Methods:**

Resting-state functional magnetic resonance imaging and graph theory analyses were used to investigate the alterations of the functional connectome in 66 SCD individuals and 64 healthy controls (HC). Pearson correlation analysis was computed to assess the relationships among network metrics, neuropsychological performance and pathological biomarkers. Finally, we used the multiple kernel learning-support vector machine (MKL-SVM) to differentiate the SCD and HC individuals.

**Results:**

SCD individuals showed higher nodal topological properties (including nodal strength, nodal global efficiency and nodal local efficiency) associated with amyloid-β levels and memory function than the HC, and these regions were mainly located in the default mode network (DMN). Moreover, increased local and medium-range connectivity mainly between the bilateral parahippocampal gyrus (PHG) and other DMN-related regions was found in SCD individuals compared with HC individuals. These aberrant functional network measures exhibited good classification performance in the differentiation of SCD individuals from HC individuals at an accuracy up to 79.23%.

**Conclusion:**

The findings of this study provide insight into the compensatory mechanism of the functional connectome underlying SCD. The proposed classification method highlights the potential of connectome-based metrics for the identification of the preclinical stage of AD.

## Background

Alzheimer’s disease (AD), the most common form of dementia, places a huge burden on modern society [1]. Unfortunately, there is presently no approved effective treatment that can stop or slow the progression of AD. It is already widely believed that the most effective treatment for AD will require intervention in the early stage of the disease, even before clinical symptoms [2]. Emerging evidence indicates that subjective cognitive decline (SCD), referring to self-reported cognitive decline in the absence of objective cognitive impairment, might serve as the typical preclinical stage along the AD continuum [3]. The risk for SCD individuals to convert to mild cognitive impairment (MCI) or AD is 4.5–6.5 times higher than that for normally ageing individuals [4–6]. Therefore, a major goal is how to identify participants with SCD in an appropriate way.

Resting-state functional magnetic resonance imaging (rs-fMRI) is a promising approach to characterize and predict the progression of disease, and functional network measures (including connectivity and topological properties) are emerging as potential intermediate biomarkers for SCD. Chiesa and colleagues focused on the alterations of the basal forebrain networks associated with AD-related pathological biomarkers in individuals with SCD. Their research indicated that lower posterior basal forebrain functional connectivity in the thalamus and the hippocampus was correlated with higher global amyloid-β (Aβ) load and contributed to understanding the pathophysiological link between cholinergic dysfunction and Aβ accumulation in the preclinical stages of AD [7]. The DZNE-Longitudinal Cognitive Impairment and Dementia (DELCODE) study further demonstrated that lower Aβ_42_ levels in SCD individuals were closely related to the perceived decline in memory and language performance [8]. In a series of functional neuroimaging studies, Wang et al. reported that the SCD group showed reduced default mode network (DMN) connectivity in the right hippocampus relative to the healthy controls (HC) [9]. According to the study by Dillen et al., higher functional connectivity from the retrosplenial cortex to the frontal cortex was observed in individuals with SCD than in the HC group [10]. Recently, from the perspective of topological property, the SCD individuals exhibited lower degree centrality in the inferior parietal region and higher degree centrality in the bilateral hippocampus and left fusiform gyrus than healthy controls [11]. However, these above findings were complicated by the fact that the different research teams used different methods and strategies. To date, no study has explored the altered functional network measures related to pathological biomarkers by combining connectivity and topological properties at the whole-brain and regional levels in SCD individuals.

If SCD individuals who are in the early stage of AD can be identified, they could potentially benefit from early targeted intervention. With the development of neuroimaging, many studies have focused on identifying brain functional alterations associated with the AD continuum, which could potentially be considered a biomarker of AD pathology. However, most of the above findings were primarily obtained based on group-level comparisons, which limited individual classification [12, 13]. To overcome this limitation, machine-learning methods combining rs-fMRI have been used in the early diagnosis of AD in recent years, and they have shown tremendous potential in individual-based disease diagnosis [14, 15]. Khazaee et al. applied topological measures as discriminating features to efficiently differentiate AD patients from healthy individuals with high accuracy [16]. In a subsequent study, Khazaee and colleagues further demonstrated that topological measures of DMN-related regions achieved great performance in the differentiation of individuals with MCI from HC [17]. Moreover, Jie et al. proposed a novel connectivity-based framework integrating multiple topological properties of functional networks to improve the classification performance of MCI individuals and healthy elderly individuals [18]. Together, previous studies have applied machine-learning techniques to investigate brain functional networks for AD or MCI diagnosis. However, it remains to be established whether machine-learning methods combining rs-fMRI play important roles in the differentiation of individuals with SCD from HC.

Here, we explored the association of altered functional connectivity and topological properties of the brain functional connectome with pathological biomarkers derived from SCD individuals obtained from the Alzheimer’s Disease Neuroimaging Initiative (ADNI) database (http://adni.loni.usc.edu). Furthermore, we combined machine-learning techniques with functional network measures (including connectivity and topological properties) to distinguish individuals with SCD from HC. This study may provide insight into understanding the pathophysiological mechanisms underlying SCD and provide potential quantitative neuroimaging biomarkers for SCD diagnosis.

## Methods

### Alzheimer’s Disease Neuroimaging Initiative

Data used in the preparation of this paper were obtained from the ADNI database (http://adni.loni.usc.edu). The ADNI was initially launched in 2003 (ADNI-1), headed by Principal Investigator Michael W. Weiner, VA Medical Center and University of California-San Francisco. The primary aim of the ADNI has been to test whether neuroimaging, biological markers and neuropsychological assessment could support the early diagnosis and track the progression of AD. For more information, see http://www.adni-info.org. The protocol was approved by the ADNI and informed consent was obtained in accordance with the Declaration of Helsinki.

### Participants

In this study, we included 66 SCD subjects and 64 well-matched HC from the ADNI database. The diagnostic criteria were described in the ADNI manual (http://www.adni-info.org). Briefly, HC participants had no subjective or informant-reported memory decline and normal performance on the Mini-Mental State Examination (MMSE, between 24 and 30), Clinical Dementia Rating (CDR, score = 0) and the Logical Memory (LM) Delayed Recall (adjusted for education level); SCD participants showed subjective memory concerns as evaluated by the Cognitive Change Index (CCI; total score from the first 12 items ≥16) [19], normal cognitive performance on the MMSE, CDR and LM-delayed recall, and no informant-reported complaint of memory decline. We also excluded participants who had a history of significant neurological and psychiatric illness (e.g., stroke, traumatic brain injury, depression and others).

### Clinical and neuropsychological measurement

Demographic characteristics and neurocognitive performance data were downloaded from the ADNI database (http://adni.loni.usc.edu). For the primary analyses, all participants underwent a battery of cognitive evaluations, including global cognitive function (MMSE) and memory function [the Rey Auditory Verbal Learning Test (RAVLT) total and delayed recall; LM-immediate and delayed recall]. The geriatric depression scale-15 (GDS-15) was used to identify the clinical depression (GDS-15 score > 5) and the neuropsychiatric inventory (NPI) was used to assess the neuropsychiatric symptoms.

### Apolipoprotein E genotyping

Apolipoprotein E (APOE) genotypes of participants in this study were obtained from the ADNI database (http://adni.loni.usc.edu, more details in the Supplementary material). All participants were classified as APOE +/+ (ε4/ε4), APOE +/− (ε4/ε2 and ε4/ε3) and APOE −/− (ε2/ε2, ε2/ε3 and ε3/ε3). Notably, not all participants had APOE genotype data, and detailed information is shown in Table 1.

**Table 1:** Demographic and neuropsychological data

Items	HC (***n*** = 64)	SCD (***n*** = 66)	Statistical Value	***P*** value
Age (years)	73.23 ± 6.69	71.28 ± 5.45	1.82	0.07^b^
Education (years)	16.56 ± 2.09	16.91 ± 2.13	−0.94	0.35^b^
Gender (male/female)	24/40	24/42	0.02	0.89^a^
APOE phenotypes (+/+, +/−, −/−)	62/64 (2/13/47)	58/66 (3/23/32)	5.70	0.06^a^
CSF Aβ_1–42_ (pg/mL)	25/64 (1401.04 ± 441.21)	11/66 (1284.44 ± 272.65)	0.81	0.43^b^
CSF t-tau (pg/mL)	25/64 (255.99 ± 129.08)	11/66 (185.44 ± 45.89)	1.75	0.09^b^
CSF p-tau (pg/mL)	25/64 (23.56 ± 14.02)	11/66 (16.19 ± 4.19)	1.70	0.10^b^
[^18^F] AV45 SUVRs	40/64 (1.11 ± 0.18)	34/66 (1.15 ± 0.18)	−0.91	0.37^b^
Intracranial volume (cm^3^)	1390.55 ± 175.92	1407.89 ± 132.81	−0.64	0.53^b^
Gray matter volume (cm3)	593.04 ± 61.13	604.97 ± 44.95	−1.27	0.21^b^
White matter volume (cm3)	511.48 ± 83.31	514.20 ± 63.25	−0.21	0.83^b^
Ventricular volume (cm3)	286.03 ± 55.57	288.73 ± 52.68	−0.28	0.78^b^
Hippocampal volume (cm3)	8.93 ± 0.99	8.95 ± 0.88	−0.13	0.90^b^
Left hippocampal volume (cm3)	4.45 ± 0.50	4.48 ± 0.48	−0.36	0.72^b^
Right hippocampal volume (cm3)	4.48 ± 0.52	4.47 ± 0.43	0.12	0.91^b^
GDS-15	0 (0–1)	1 (0–1)	−1.86	0.06^c^
NPI	0 (0–1)	0 (0–0.25)	−0.78	0.44^c^
CCI	–	20 (17.75–26)	–	–
MMSE	28.88 ± 1.53	29.02 ± 1.14	−0.59	0.55^b^
LM-immediate	15.28 ± 3.60	14.65 ± 3.20	1.01	0.29^b^
LM-delayed recall	11.31 ± 1.55	11.14 ± 1.58	0.64	0.52^b^
RAVLT-total	48.64 ± 9.64	46.42 ± 9.52	1.32	0.19^b^
RAVLT-delayed recall	6.31 ± 2.19	6.39 ± 2.26	−0.21	0.84^b^

No significant differences were found in the age, gender, years of education, APOE genotypes, CSF biomarkers, brain tissue volumes, psychological assessments and cognitive performance between the HC and SCD group

*Abbreviations*: *HC* Health control, *SCD* Subjective cognitive decline, *APOE* Apolipoprotein E, *CSF* Cerebrospinal fluid, *SUVR* Standardized uptake values ratio, *GDS* Geriatric depression scale, *NPI* Neuropsychiatric inventoryl, *CCI* Cognitive change index, *MMSE* Mini mental state examination, *LM* Logical Memory, *RAVLT* Rey Auditory Verbal Learning Test

Values are presented as the mean ± standard deviation and median (interquartile range)

^a^ the *p* value was obtained by χ2 test, ^b^ the *p* value was obtained by two-sample t tests, ^c^ the *p* value was obtained by Mann-Whitney tests

### Cerebrospinal fluid biomarkers

Lumbar puncture and cerebrospinal fluid (CSF) sample preparation were performed as described in the ADNI manual (http://adni.loni.usc.edu/research/protocols/biospecimens-protocols/, more details in the Supplementary material). CSF Aβ_1–42_, t-tau and p-tau were measured using INNOBIA AlzBio3 immunoassay kit-based reagents (Innotest, Fujirebio, Ghent, Belgium). Notably, not all participants had CSF sample data since lumbar puncture is an invasive operation. In this study, 11 out of 66 SCD subjects and 25 out of 64 HC subjects had CSF sample data available (Table 1).

### [^18^F] AV45 positron emission tomography scans

[^18^F] AV45 positron emission tomography (PET) data were processed as described in the standardized protocol (http://adni.loni.usc.edu/methods/, more details in the Supplementary material). Mean florbetapir standard uptake value ratios (SUVRs) were computed within these brain regions (lateral and medial anterior frontal, lateral temporal, posterior cingulate, and lateral parietal cortex) and normalized to the whole cerebellum as the reference region. In this study, 34 out of 66 SCD individuals and 40 out of 64 HC subjects had PET SUVRs data available (Table 1).

### MRI acquisition

All participants were examined on a SIEMENS 3.0-T scanner. The examination protocol included the high-resolution T1-weighted sequence [repetition time (TR) = 2300 ms, flip angle (FA) = 9°, echo time (TE) = 2.98 ms, inversion time (TI) = 900 ms, FOV = 256 × 240 mm^2^, number of slices = 176, spatial resolution = 1.2 × 1.1 × 1.1 mm^3^] and the rs-fMRI sequence [TR = 3000 ms, TE = 30 ms, number of slices = 48, slice thickness = 3.4 mm, number of volumes = 197, FOV = 220 × 220 mm^2^, spatial resolution = 3.44 × 3.44 × 3.40 mm^3^].

### Image preprocessing and network construction

Brain tissue segmentation was performed using the Computational Anatomy Toolbox (CAT12, http://www.neuro.uni-jena.de/cat/) as implemented in the Statistical Parametric Mapping analysis package (SPM12, http://www.fil.ion.ucl.ac.uk/spm/soft-ware/spm12/). The main preprocessing included correction for bias-field inhomogeneities; tissue segmentation into white matter (WM), grey matter (GM) and CSF; and spatial normalization with the DARTEL algorithm. The intracranial volume was obtained by summing the volumes of the GM, WM and CSF.

The rs-fMRI data were preprocessed by the Data Processing & Analysis for Brain Imaging (DPABI V4.1, http://rfmri.org/dpabi/). The main preprocessing steps included slice time correction, head motion correction (six head motion parameters), normalization to the Montreal Neurological Institute (MNI) space (EPI template with 3 mm isotropic voxels), filtering (0.01–0.1 Hz) and multiple linear regression analysis (including the Friston 24 parameters, cerebrospinal fluid and white matter signals). Participants who had performed an angular rotation > 2° or a displacement > 2 mm in any direction were excluded. In addition, the two groups did not show significant differences in the mean frame-wise displacement (FD) suggested by Jenkinson et al. [20]. To define the network nodes, an automated anatomical labeling (AAL) atlas was performed to divide the whole brain into 90 regions of interest (ROIs) (the abbreviations in Supplemental Table 1). To define the network edge, we calculated the Pearson correlation of the regional mean time series between each pair of 90 ROIs. To further remove spurious correlations, only those correlation coefficients whose corresponding *p* values were lower than a statistical threshold (*p* < 0.05, Bonferroni-corrected) were retained [21].

### Network analysis

#### Network topological analyses

The topological properties of network were analyzed using the Graph Theoretical Network Analysis Toolbox (GRETNA, http://www.nitrc.org/projects/gretna/). We evaluated the global properties of brain network by the following measures: network strength, clustering coefficient, shortest path length, small-worldness, global efficiency, local efficiency, hierarchy and assortativity. In addition, we used nodal strength, nodal clustering coefficient, nodal shortest path length, nodal global efficiency and nodal local efficiency to describe the regional properties of the functional network. The details on the definitions and mathematical equations of these parameters are presented in the Supplementary material.

### Hub distribution

Based on the individual weighted functional network, we computed the rich club coefficient and normalized the rich club coefficient for each participant [22]. Normalized rich club coefficients higher than 1 over a range of thresholds showed the existence of rich club organization in the brain network. To identify the hub distribution of the functional network, the top 14 (15%) brain regions with the highest nodal degree across all participants were defined as rich club regions [23, 24]. On the basis of the hub and non-hub regions, the connections of the network were grouped into rich club connections (between hub nodes and hub nodes), feeder connections (between hub nodes and non-hub nodes), and local connections (between non-hub nodes and non-hub nodes) (Fig. 2c) [25–27]. In addition, to confirm the stability of our results, we estimated the rich club, feeder and local connections based on the top 10 and 20% node degree, respectively (more details in the Supplementary material, Supplemental Figure 1). The rich-club analysis was performed using GRETNA toolbox.

### Measures of connection distance

The physical connection distance between the 2 functionally connected brain regions was estimated as the Euclidean distance (${d}_{ij}=\sqrt{{\left({x}_i-{x}_j\right)}^2+{\left({y}_i-{y}_j\right)}^2+{\left({z}_i-{z}_j\right)}^2}$, where *x*, *y*, and *z* are the stereotactic coordinates of the centroid of each node) [28]. The distance threshold was chosen through the identification of the shortest and longest possible distance between 2 nodes and the division of the difference into 3 equal ranges (short 7.61–55.4 mm, medium 55.4–103.19 mm, and long 103.19–150.98 mm) [29].

### Statistical analysis

Differences between the HC group and the SCD group in demographic, neuroimaging characteristics and cognitive performance were assessed using two-sample *t* test, Mann-Whitney test or a chi-squared (χ^2^) test by Statistical Package for Social Sciences (SPSS V22). The significance level was set at *p < 0.05*.

The global properties of brain network were compared by two-sample *t* test between the HC group and the SCD group (*p < 0.05*, uncorrected). To determine the regions with significantly altered topological properties, two-sample *t-*tests were performed on the nodal properties by false-discovery rate correction (*q* = 0.05). The connectivity strength of rich club, feeder and local connections between the HC group and the SCD group were compared by two-sample *t-*tests (*p < 0.05*, uncorrected).

To localize the specific component (i.e., subnetwork) that had significantly different connectivity strength between the HC group and the SCD group, we used a network-based statistic (NBS) approach [30]. Briefly, a primary threshold (*p* < 0.001, uncorrected) was applied to the two-sample *t* test computed for each connection to define a set of suprathreshold connections among which any connected subnetworks and their size were then determined. A corrected *p* value was computed for each connected component using the null distribution of the maximal component size (i.e., the number of links), which was empirically derived by using a non-parametric permutation approach (5000 permutations).

We performed Pearson correlation analyses to investigate the relationships between altered network metrics (functional connections and topological properties), pathological makers and neuropsychological performance (*p < 0.05*, uncorrected).

### Multiple kernel support vector machine

Apart from revealing altered functional connectivities and topological properties of functional networks in the SCD group, we also used these two kinds of features to accurately differentiate the SCD individuals from the HC group. We employed the original functional network characteristics including 4005 (90 × 89/2) functional connections and 270 (90 edge strength, 90 nodal global efficiency and 90 nodal local efficiency) nodal properties, as features for subsequent analyses. As in the previous study, the two-sample *t* test was used to select features (*p* < 0.001, uncorrected) [31, 32]. Then, the discriminant analysis was performed by using the support vector machine (SVM) as a classifier. To further integrate the complementary information of these two kinds of network metrics (functional connections and nodal properties), we employed the multiple kernel learning SVM (MKL-SVM) to fuse these features as described in other studies [33, 34]. In the current study, the leave-one-out cross-validation (LOOCV) strategy was used to assess the classification performance. The performance of a classifier could be quantified using accuracy, sensitivity, specificity and the area under the receiver operating characteristic (ROC) curve (AUC). Note that the specificity represented the proportion of the HC individuals correctly predicted, while the sensitivity represented the proportion of the SCD individuals correctly predicted. Accuracy is defined as (TP + TN)/(TP + TN + FN + FP), sensitivity is defined as TP/(TP + FN) and specificity is defined as TN/(FP + TN), where TN is the number of true negatives (number of HC individuals correctly classified), TP is the number of true positives (number of SCD individuals correctly classified), FN is the number of false negatives (number of SCD individuals classified as HC individuals), and FP is the number of false positives (number of HC individuals classified as SCD individuals). In addition, the AUC is an evaluation measure based on the ROC curve, which illustrates the performance of the classifier. The ROC curve is delineated by plotting 1-specificity and sensitivity at different thresholds.

## Results

### Demographic and clinical characteristics

Demographic and clinical data for the HC group and SCD group are summarized in Table 1. No significant differences were found in the age, gender, years of education, APOE genotypes, CSF biomarkers, brain tissue volumes, psychological assessments and cognitive performance between the HC and SCD group.

### Alterations of topological properties

First, we compared the global topological properties of the whole brain between the HC and SCD group (Table 2). We found significantly increased network strength, clustering coefficient, global efficiency and local efficiency in the SCD group compared with the HC group (*P* < 0.05, uncorrected). Furthermore, the shortest path length and small-worldness in the SCD group was significantly lower than that in the HC group (*P* < 0.05, uncorrected). No statistical significance was observed in the hierarchy coefficient and assortativity coefficient between HC and SCD group.

**Table 2:** Global properties of functional network in HC and SCD

Global properties	HC	SCD	***P*** value
Network strength	13.13 ± 3.21	14.90 ± 3.02	0.002*
Clustering coefficient	0.33 ± 0.04	0.35 ± 0.03	0.001*
Shortest path length	3.37 ± 0.34	3.20 ± 0.28	0.001*
Small-worldness	1.12 ± 0.15	1.05 ± 0.09	0.002*
Global efficiency	0.30 ± 0.03	0.31 ± 0.03	0.001*
Local efficiency	0.32 ± 0.02	0.33 ± 0.02	0.002*
Hierarchy	0.02 ± 0.13	−0.001 ± 0.13	0.387
Assortativity	−0.04 ± 0.11	−0.09 ± 0.16	0.067

Significantly increased network strength, clustering coefficient, global efficiency and local efficiency in the SCD group compared with the HC group (*P* < 0.05, uncorrected). The shortest path length and small-worldness in the SCD group was significantly lower than that in the HC group (*P* < 0.05, uncorrected). No statistical significance was observed in the hierarchy coefficient and assortativity coefficient between HC and SCD group

*Abbreviations*: *HC* Health control, *SCD* Subjective cognitive decline

*indicates a statistical difference between groups, *p* < 0.05

**Fig. 1:**
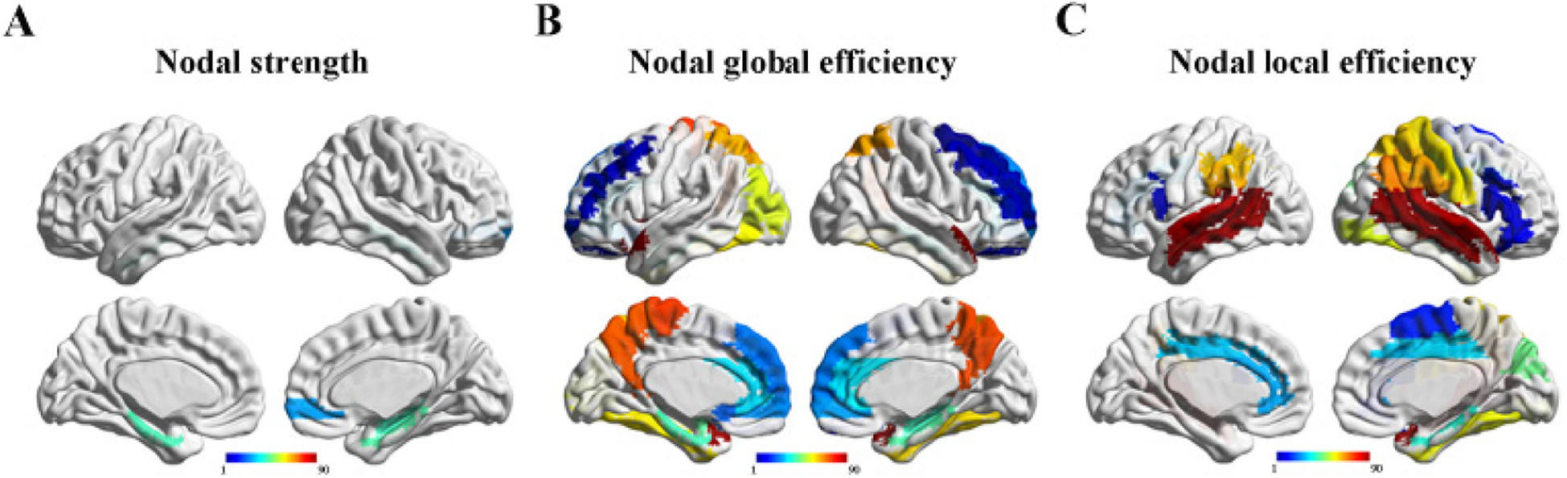
The altered nodal strength, nodal global efficiency and nodal local efficiency between SCD and HC. **a** The SCD group showing significantly increased strength in four brain regions; **b** The SCD group showing significantly increased nodal global efficiency in 28 brain regions; **c** The SCD group showing significantly increased nodal local efficiency in 27 brain regions. Abbreviations: SCD, subjective cognitive decline; HC, healthy control; The color bar represents the label of brain regions in AAL-90 atlas

Second, the nodal properties including the nodal strength, nodal clustering coefficient, nodal shortest path length, nodal global efficiency and nodal local efficiency were compared for each brain region. The SCD group showed significantly increased nodal strength in the right superior frontal gyrus and the bilateral medial temporal lobe (*P* < 0.05, FDR corrected, Fig. 1a, Supplemental Table 2). The increased nodal global efficiency and nodal local efficiency mainly in the frontal, temporal and parietal regions were found in the SCD group (*P* < 0.05, FDR corrected, Fig. 1a and b, Supplemental Table 2). Additionally, these brain regions were mainly distributed in the DMN. Previous studies demonstrated that the nodal global efficiency and nodal local efficiency are closely related to the nodal shortest path length and nodal clustering coefficient [22, 35–37]. The inverse of nodal shortest path length and nodal clustering coefficient are the reasonable approximation of nodal global efficiency and nodal local efficiency, respectively, when there are no huge differences among the distances in the connected network. By contrast, the efficiency measures are more adoptable for the real networks and also more applicable to disconnected networks [36, 37]. Thus, our study mainly focused on the nodal strength, nodal global efficiency and nodal local efficiency, and the results of the nodal shortest path length and nodal clustering coefficient were described in the Supplementary material, Supplemental Figure 2 and Supplemental Table 3.

### Group differences in rich club organization

**Fig. 2:**
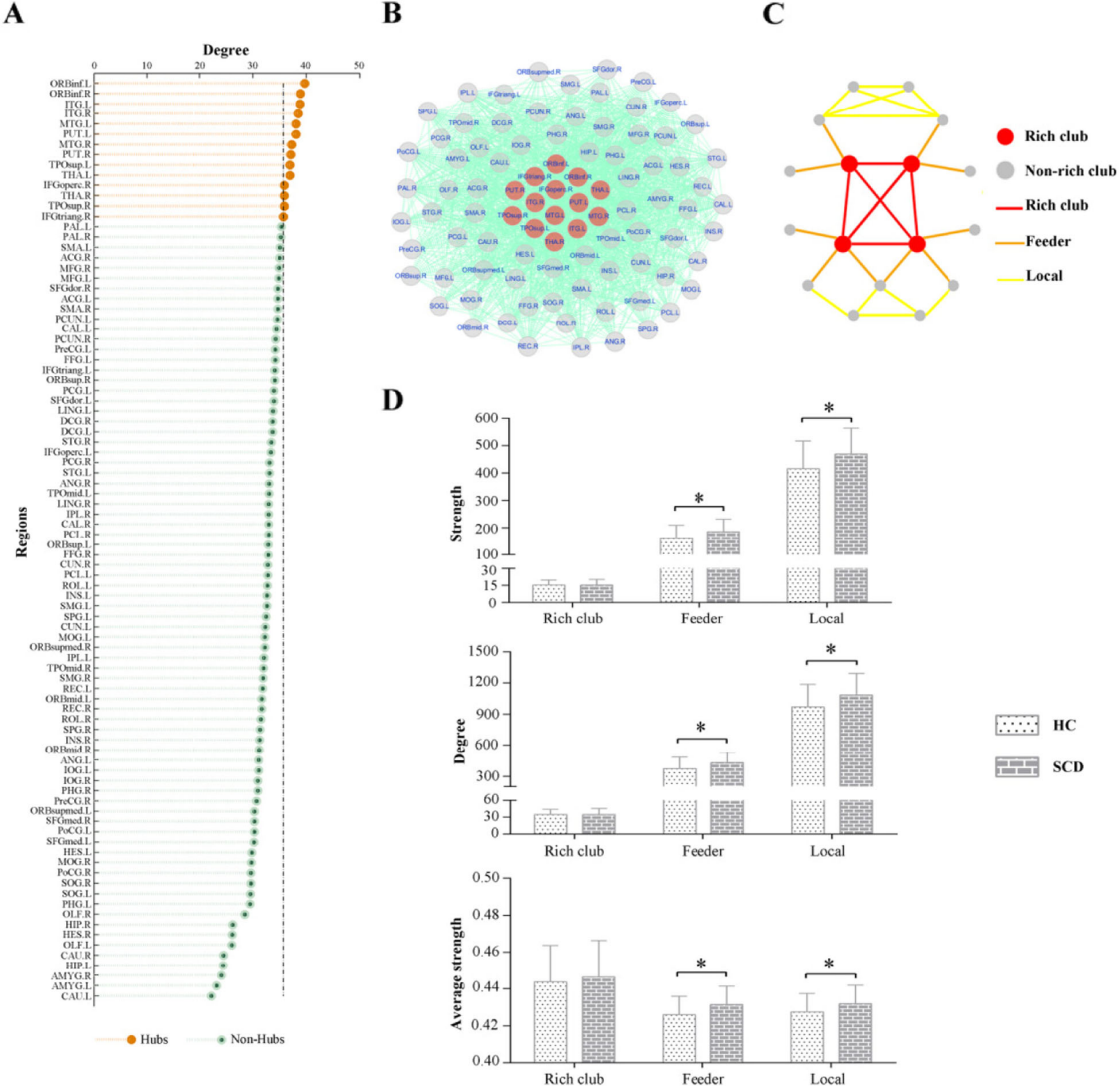
The altered rich club organization between SCD and HC. **a** and **b** The top 14 (15%) highest-degree nodes were chosen to represent rich club nodes based on the averaged nodal degree across all participants; **c** The sketch map of rich club organization; **d** Significant differences in the strength, degree and average strength of the feeder and local connections were identified, while no significant differences were found in rich club connections. Abbreviations: SCD, subjective cognitive decline; HC, healthy control; * indicates a statistical difference between groups, *p* < 0.05

The top 14 (15%) highest-degree nodes were chosen to represent rich club nodes based on the averaged nodal degree across all participants (Fig. 2a and b, orange nodes). The remaining nodes were identified as peripheral regions. Moreover, significant differences in the strength, degree and average strength of the feeder and local connections were identified, while no significant differences were found in rich club connections (Fig. 2d). In detail, the SCD group exhibited higher connections compared with the HC group (strength: feeder *p* = 0.002; local *p* = 0.002; degree: feeder *p* = 0.003; local *p* = 0.003; average strength feeder *p* = 0.028; local *p* = 0.045).

### Group differences in functional connectivity based on the NBS approach

**Fig. 3:**
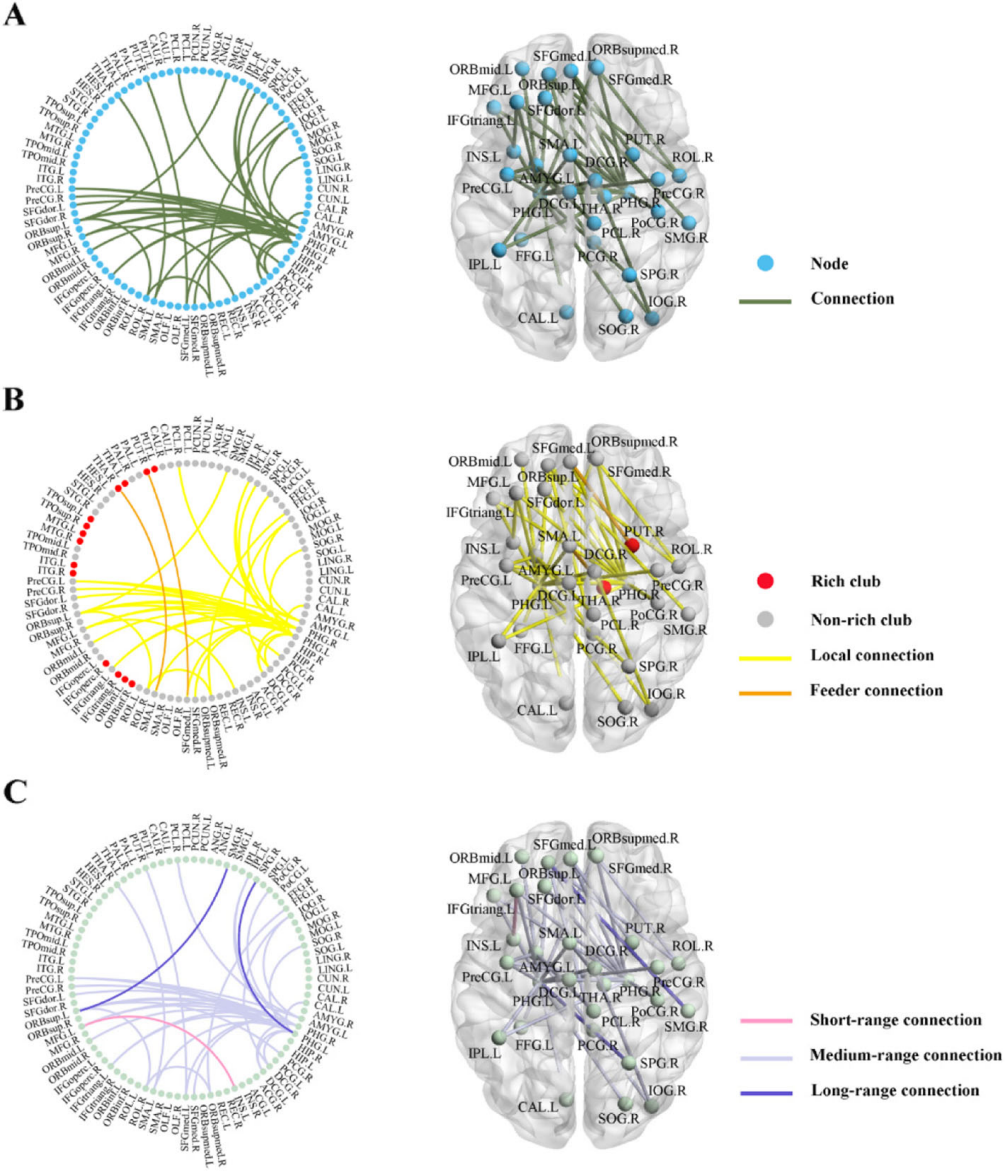
The altered connected subnetwork based on the NBS analysis. A single connected subnetwork with 30 nodes and 35 connections, which exhibited higher connection strength in the SCD group compared with the HC group (*p* < 0.001, corrected); **b** The 28 out of 30 node within the subnetwork were classified into non-hub regions and the 33 out of 35 connections belonged to the local connections; **c** The increased connectivity was primarily involved in the medium-range connections based on the Euclidean distance. Abbreviations: SCD, subjective cognitive decline; HC, healthy control; NBS, network-based statistic

Using the non-parametric NBS analysis, a single connected subnetwork with 30 nodes and 35 connections exhibited higher connection strength in the SCD group than in the HC group (*p* < 0.001, corrected) (Fig. 3a and Supplemental Table 4). These increased connections were composed mainly of inter-region connections, which linked the bilateral parahippocampal gyrus (PHG) with the frontal gyrus, cingulate and paracingulate gyri and parietal regions (23/35, 65.7%). Interestingly, 28 out of 30 nodes within the subnetwork were classified into non-hub regions and 33 out of 35 connections belonged to the local connections (between non-hub nodes and non-hub nodes) (Fig. 3b). In addition, the increased connectivity was primarily involved in the medium-range connections (33/35, 94.3%) based on the Euclidean distance (Fig. 3c).

### Relationships among altered network metrics, biomarkers and neuropsychological performance

**Fig. 4:**
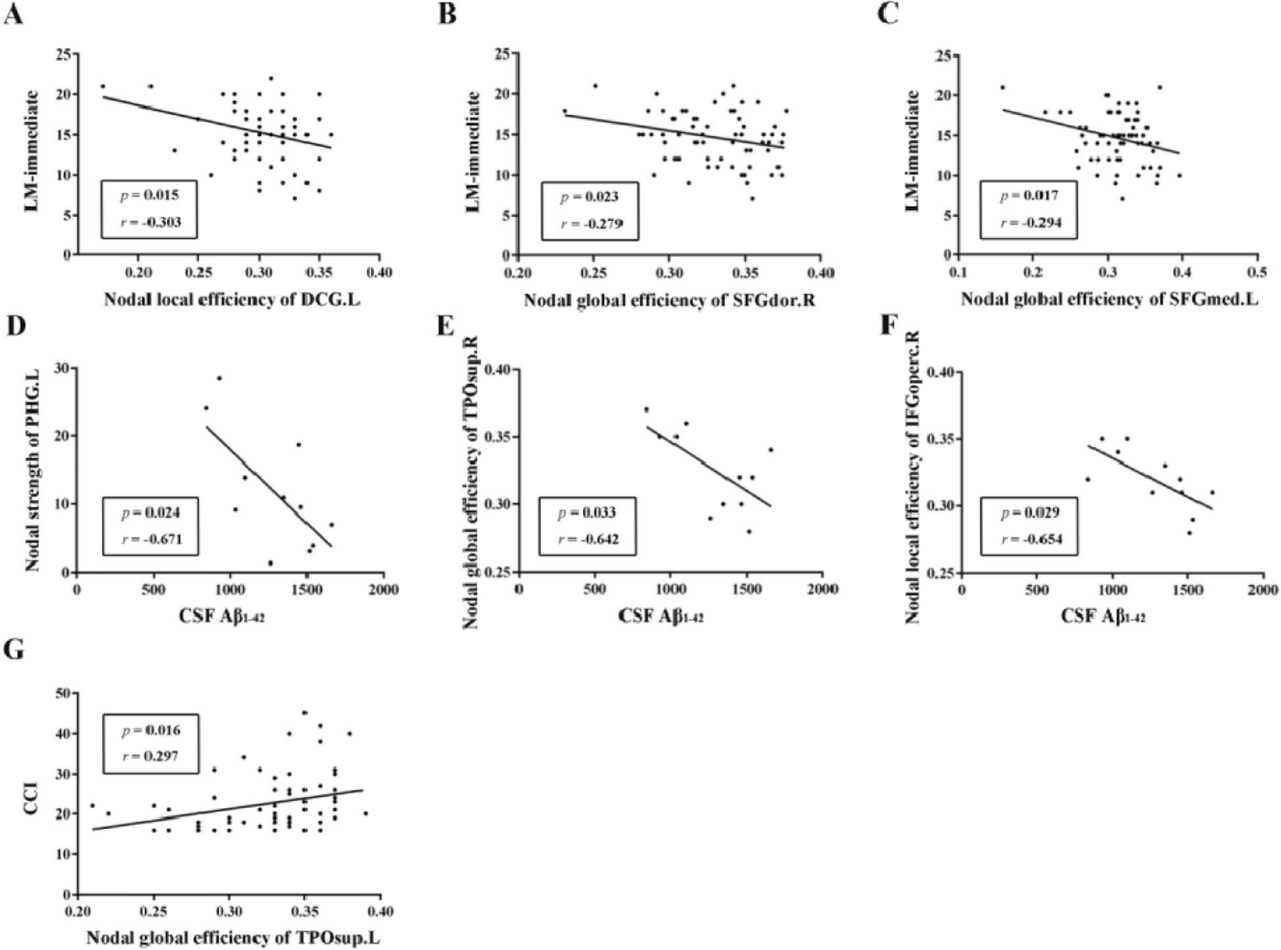
Relationships among altered network metrics, biomarkers and neuropsycholohical performance. **a** The scores on the LM-immediate were negatively associated with nodal local efficiency of the DCG.L (*r* = − 0.303, *P* = 0.015) in the HC group; **b** and **c** The scores on the LM-immediate were negatively associated with nodal global efficiency of the SFGdor.R (*r* = − 0.279, *P* = 0.023) (**b**) and the SFGmed.L (*r* = − 0.294, *P* = 0.017) (**c**) in the SCD group; **d**, **e** and **f** The CSF Aβ1–42 was negatively related to the nodal strength of PHG.L (*r* = − 0.671, *P* = 0.024) (**d**), nodal global efficiency of the TPOsup.R (*r* = − 0.642, *P* = 0.033) (**e**) and nodal local efficiency of the IFGoperc.R (*r* = − 0.654, *P* = 0.029) (**f**) in the SCD group; **g** The scores on the CCI were positively associated with nodal global efficiency of the TPOsup.L (*r* = 0.297, *P* = 0.016) in the SCD group. Abbreviations: HC, healthy control; SCD, subjective cognitive decline; DCG.L, left median cingulate and paracingulate gyri; SFGdor.R, right dorsolateral superior frontal gyrus; SFGmed.L, left medial superior frontal gyrus; PHG.L, left parahippocampal gyrus; TPOsup.R, right temporal pole-superior temporal gyrus; IFGoperc.R, right inferior frontal gyrus-opercular part; CSF, cerebrospinal fluid; Aβ, amyloid-β; TPOsup.L, left temporal pole-superior temporal gyrus; LM, Logical Memory; CCI, cognitive change index

We performed Pearson correlation analyses to investigate the relationships between altered network metrics (functional connections and nodal properties), pathological markers and neuropsychological performance in the HC and SCD group, respectively. In the HC group, the scores on the LM-immediate was significantly negatively correlated with nodal local efficiency of the left median cingulate and paracingulate gyri (DCG.L) (*r* = − 0.303, *P* = 0.015, uncorrected) (Fig. 4a). However, no significant relationship was detected between AD pathological markers and altered network metrics in the HC group. In the SCD group, we found that the scores on the LM-immediate were negatively associated with nodal global efficiency of the right dorsolateral superior frontal gyrus (SFGdor.R) (*r* = − 0.279, *P* = 0.023, uncorrected) (Fig. 4b) and the left medial superior frontal gyrus (SFGmed.L) (*r* = − 0.294, *P* = 0.017, uncorrected) (Fig. 4c). In addition, the CSF Aβ_1–42_ was negatively related to the strength of the PHG.L (*r* = − 0.671, *P* = 0.024, uncorrected) (Fig. 4d), the nodal efficiency of the right temporal pole-superior temporal gyrus (TPOsup.R) (*r* = − 0.642, *P* = 0.033, uncorrected) (Fig. 4e) and the nodal local efficiency of the right inferior frontal gyrus-opercular part (IFGoperc.R) (*r* = − 0.654, *P* = 0.029, uncorrected) (Fig. 4f). We also detected the relationships between the CSF Aβ_1–42_ and the nodal strength of PHG.L, nodal global efficiency of the TPOsup.R and nodal local efficiency of the IFGoperc.R (Supplemental Figure 3). Furthermore, we found that the CCI was positively related to the nodal global efficiency of TPOsup.L in the SCD group (*r* = 0.297, *P* = 0.016, uncorrected) (Fig. 4g).

### Discriminative analysis

**Fig. 5:**
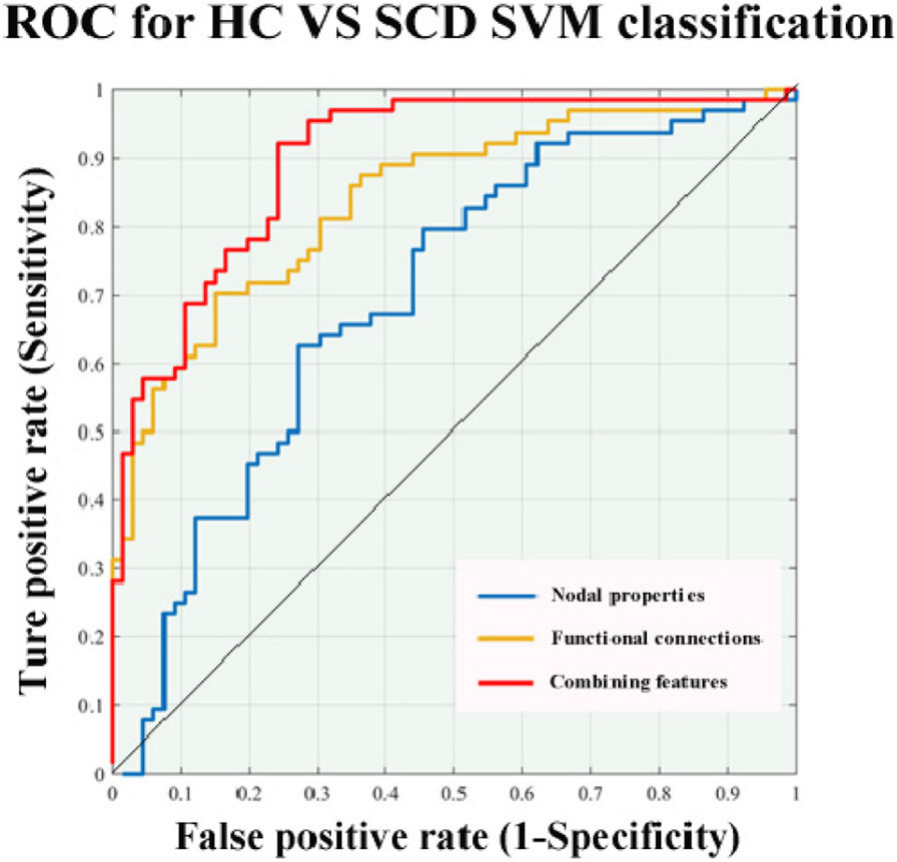
Result of discriminative analysis. For single-modality analyses, the functional connections exhibited the higher accuracy rate (76.15%) than the nodal properties which achieved the accuracy rate of 66.15%. Typically, classification accuracy improved after combining the network features of the two modalities, achieving the accuracy of up to 79.23%

In this study, nodal properties and connections were utilized to classify whether a sample belonged to the SCD group (Fig. 5, Table 3 and Supplemental Table 5). For single-modality analyses, the functional connections exhibited a higher accuracy rate (76.15%) than the nodal properties which achieved an accuracy rate of 66.15%. Typically, classification accuracy improved after combining the network features of the two modalities, achieving an accuracy of up to 79.23%.

**Table 3:** Results of the discrimination analyses derived from the SVM between HC and SCD

Feature	Accuracy (%)	Sensitivity (%)	Specificity (%)	AUC (%)
**Nodal properties**	66.15	64.06	68.18	69.82
**Functional connections**	76.15	70.31	81.82	84.02
**Combining features**	79.23	73.44	84.85	89.77

For single-modality analyses, the functional connections exhibited a higher accuracy rate (76.15%) than the nodal properties which achieved an accuracy rate of 66.15%. The classification accuracy improved after combining the network features of the two modalities, achieving an accuracy of up to 79.23%

*Abbreviations*: *HC* Health control, *SCD* Subjective cognitive decline, *AUC* The area under the curve, *SVM* Support vector machine

## Discussion

In this study, we investigated the connectivity and topological alterations of brain functional connectome related to pathological biomarkers and their diagnostic value in individuals with SCD. Higher nodal topological properties (including nodal strength, nodal global efficiency and nodal local efficiency) associated with CSF Aβ_1–42_ levels were found in the SCD individuals than in the HC, mainly located in DMN-related brain regions. Moreover, SCD individuals showed increased local and medium-range connectivity mainly between the bilateral PHG and other DMN-related regions relative to the HC. These enhanced functional network measures may reflect a compensatory mechanism that preserves memory performance in SCD individuals. Importantly, the aberrant functional network measures exhibited good classification performance in differentiating SCD individuals from healthy controls.

SCD is a stage of mild neuronal injury but with clinically normal cognitive performance remaining that contributes to sufficient functional compensation [11, 38]. Here, we found that increased nodal properties, including nodal strength, nodal global efficiency and nodal local efficiency in the SCD group were mainly located in the frontal, medial temporal, parietal and precuneus cortices, which were distributed in the DMN. The DMN, involved in self-reflection and memory processes, is thought to be the most vulnerable functional network in AD [39, 40]. Thus, we hypothesized that the higher topological properties in the DMN might compensate for impaired memory in SCD individuals. This finding was in line with the study by Li et al., which reported higher degree centrality in the medial temporal region in SCD individuals than in healthy controls [11]. Another study came to a similar conclusion, reporting that the SCD group showed higher nodal efficiency in the DMN-related region (e.g., parahippocampal gyrus) than the HC group [41]. However, a brain structural connectome study based on diffusion tensor imaging (DTI) indicated that disrupted topological efficiency of the prefrontal regions and thalamus was detected in SCD individuals [42]. These results suggested that SCD might be at a stage of structural damage and functional compensation in the AD continuum and findings of functional neuroimaging studies complemented the findings derived from structural neuroimaging studies.

Moreover, we found that the CSF Aβ_1–42_ level was negatively related to the strength of PHG.L, the nodal global efficiency of the TPOsup.R and the nodal local efficiency of the IFGoperc.R. Based on the arterial spin labeling (ASL) technique, reduced regional cerebral blood flow (rCBF) was observed in the temporal regions of MCI patients, but increased rCBF was found along the midline DMN regions in SCD individuals compared to healthy elderly individuals [43]. Interestingly, Perrotin et al. observed increased Aβ deposition, determined by PET, in the DMN region (e.g., prefrontal regions, cingulate cortex and precuneus) related to SCD subjects’ reduced confidence relative to that observed in the HC group [44]. Taken together, the enhanced topological properties in DMN-related regions may reflect the compensatory mechanism associated with increased rCBF in response to Aβ accumulation.

In addition to the enhanced topological properties in the DMN, we found that SCD individuals also showed increased local and medium-range connectivity mainly between bilateral the PHG and other DMN-related regions compared to the HC. There were three characteristics summarized from this finding. First, the increased connectivity in SCD individuals was mainly between the bilateral PHG and other DMN-related regions. This prior study demonstrated that the presence of cognitive complaints, which was the primary diagnostic point of SCD, was related to cortical atrophy in the PHG caused early by AD neuropathology [45, 46]. Using the fluorodeoxyglucose-PET (FDG-PET), Mosconi et al. concluded that SCD individuals exhibited lower metabolic rates for glucose in the PHG, inferior parietal lobe, inferior frontal gyrus and other regions than HC, and the greatest SCD-related reduction was observed in the PHG [47]. Evidence from the task-state fMRI study showed that SCD individuals with increased activation in the PHG showed normal performance during the divided attention condition task [48]. Recently, the only longitudinal and placebo-controlled trial indicated that SCD participants who received the ganglioside had higher functional connectivity over the DMN regions associated with improved working memory performance than those who did not [49]. Accordingly, the increased connectivity between the PHG and other DMN-related regions suggested that there may be compensatory mechanisms in individuals with SCD that allowed them to preserve clinically normal cognitive function.

Second, the increased connectivity in SCD individuals was mainly classified into local connectivity linking non-hub nodes to non-hub nodes. Jones et al. proposed a cascading network deterioration in AD: the dysfunction begins with a local overload and then transfers a processing burden to the other systems that include prominent connectivity hubs, eventually resulting in widespread system failures [50]. Evidence from the brain structure connectome found that connectivity among non-hub nodes was disrupted but rich-club connectivity remained stable in SCD individuals relative to MCI and AD patients [23]. Similar findings were presented by Daianu et al., which indicated predominant disruptions in the peripheral network components in the early stage of AD [51]. Due to rich club organization, hub nodes are more densely connected among themselves than with non-hub nodes. A resting-state neuroimaging study has hypothesized that the local hyperconnectivity in SCD individuals is a result of brain plasticity after damage to the neural network [11]. Therefore, we deduced that local connectivity had more sufficient compensation ability than rich-club connectivity in the preclinical stage of AD. Third, the increased connectivity was primarily involved in the medium-range connections based on the Euclidean distance. Direct evidence suggested that long-range connections could provide quick links among remote brain regions and play a crucial role in maintaining normal cognitive function [52]. In addition, the metabolic costs of brain regions are closely related to inter-regional connectivity distance: long-range connections consume more energy than short-range connections [28]. Dai et al. reported that impaired long-range connections underlie the cognitive impairments in AD patients [53]. Hence, we speculated that increased medium-range connections in SCD individuals might reflect the balance of metabolic costs and information processing.

The identification of objective functional neuroimaging biomarkers is urgently needed because it could assist clinical decisions for individuals. To date, SCD diagnosis mainly depends on the various psychological scales. In a previous study, Zhang et al. used MRI, CSF biomarkers and FDG-PET to differentiate MCI individuals from HC and achieved a classification accuracy of 76.4% [54]. Due to the invasive operation, this approach was probably not good for SCD individuals who had normal cognition. We combined connectivity and topological properties of the functional connectome and then applied the MKL-SVM framework to differentiate SCD individuals from HC. We found that classification accuracy improved after combining the network features of the two modalities, achieving an accuracy of up to 79.23% and increased functional connectivity between the PHG and DMN-related regions showed the most discriminative ability. Recently, Yan et al. employed structural and functional connectivity to differentiate SCD individuals from HC, and it exhibited good classification performance [55]. Compared to the classification performance of single-modal methods (fMRI: 77.81%; DTI: 58.51%), multimodal analyses exhibited the higher accuracy rate (80.24%). However, this study ignored higher-order interactions (i.e., topological properties) of many brain regions working together, which might influence the performance of the classifier.

Although our study tried to propose a new perspective for understanding the aberrant functional network architecture and the early identification of SCD, a few limitations still require future study. First, the topological organization of the brain functional network is affected by different parcellation strategies. Other brain-wide graphs may be used to further assess the reliability in the differentiation of SCD individuals. Second, this study was performed on a small sample size of SCD individuals with pathological markers, and we look forward to expanding the sample size to validate our results in future studies. A multicentre longitudinal study is essential, and an individualized predictive system for disease progression in SCD individuals will be formulated in the future. Third, some other analysis methods of graph theory (for example, Minimum Spanning Tree) were not included in this study and we will apply these new methods in future studies [56, 57]. Fourth, no significant results survived after FDR or Bonferroni correction in correlation analyses. To explore their relationships, we didn’t perform correction for multiple comparisons. Last, we compared only SCD and HC individuals; we did not involve MCI and AD patients. We added those participants in the subsequent study.

## Conclusion

This study demonstrates a compensatory mechanism of DMN-related connectivity and topological properties in the functional connectome in SCD individuals. Our findings provide novel insights into the pathophysiological mechanism of SCD and highlight the potential for applying connectome-based metrics as diagnostic biomarkers.

## Supplementary information

**Additional file 1: Supplemental Fig. 1.** Altered connections illustrated by rich club, feeder and local connections based on different thresholds. A. the top 10% node degree as threshold; B. the top 20% node degree as threshold. Abbreviations: SCD, subjective cognitive decline; HC, healthy control. * indicates a statistical difference between groups, *p* < 0.05.

**Additional file 2: Supplemental Fig. 2**. The altered nodal shortest path length and nodal clustering coefficient between SCD and HC. A. The SCD group showing significantly decreased nodal shortest path length in thirteen brain regions; B. The SCD group showing significantly increased nodal clustering coefficient in sixteen brain regions; Abbreviations: SCD, subjective cognitive decline; HC, healthy control; The color bar represents the label of brain regions in AAL-90 atlas.

**Additional file 3: Supplemental Fig. 3.** Relationships between altered network metrics and biomarkers in the HC group. No significance was found between the CSF Aβ_1–42_ and the nodal strength of PHG.L (*r* = 0.197, *P* = 0.345) (A), nodal global efficiency of the TPOsup.R (*r* = 0.069, *P* = 0.744) (B) and nodal local efficiency of the IFGoperc.R (*r* = − 0.046, *P* = 0.826) (C) in the HC group. Abbreviations: HC, healthy control; PHG.L, left parahippocampal gyrus; TPOsup.R, right temporal pole-superior temporal gyrus; IFGoperc.R, right inferior frontal gyrus-opercular part; CSF, cerebrospinal fluid; Aβ, amyloid-β.

**Additional file 4: Supplemental Table 1.** Brain areas and their abbreviations in the AAL-90 atlas.

**Additional file 5: Supplemental Table 2.** The comparison of nodal properties between HC and SCD. The SCD group showed significantly increased nodal strength in the right superior frontal gyrus and the bilateral medial temporal lobe (*P* < 0.05, FDR corrected). The increased nodal global efficiency and nodal local efficiency mainly in the frontal, temporal and parietal regions were found in the SCD group (*P* < 0.05, FDR corrected). Abbreviations: SCD, subjective cognitive decline; HC, healthy control.

**Additional file 6: Supplemental Table 3.** The comparison of nodal shortest path length and nodal clustering coefficient between HC and SCD. The results of the nodal shortest path length and nodal clustering coefficient were described in this table. Abbreviations: SCD, subjective cognitive decline; HC, healthy control.

**Additional file 7: Supplemental Table 4.** The subnetwork derived from NBS analysis. A single connected subnetwork with 30 nodes and 35 connections exhibited higher connection strength in the SCD group than in the HC group (*p* < 0.001, corrected). Abbreviations: SCD, subjective cognitive decline; HC, healthy control.

**Additional file 8: Supplemental Table 5.** The features selected by SVM for HC VS SCD classification. Nodal properties and connections were utilized to classify whether a sample belonged to the SCD group. Abbreviations: SCD, subjective cognitive decline; HC, healthy control; SVM, support vector machine.

**Additional file 9: Supplementary materials legends**. Details regarding methods and materials.

## Data Availability

The data used during this study are available from the ADNI (http://adni.loni.usc.edu).

## Abbreviations

SCD: Subjective cognitive decline
AD: Alzheimer’s disease
MCI: Mild cognitive impairment
rs-fMRI: Resting-state functional magnetic resonance imaging
Aβ: Amyloid-β
DMN: Default mode network
HC: Healthy controls
ADNI: Alzheimer’s Disease Neuroimaging Initiative
MMSE: Mini-Mental State Examination
CDR: Clinical dementia rating
LM: Logical Memory
GDS: Geriatric depression scale
NPI: Neuropsychiatric inventoryl
CCI: Cognitive Change Index
RAVLT: Rey Auditory Verbal Learning Test
APOE: Apolipoprotein E
CSF: Cerebrospinal fluid
PET: Positron emission tomography
SUVRs: Standard uptake value ratios
TR: Repetition time
FA: Flip angle
TE: Echo time
TI: Inversion time
CAT: Computational Anatomy Toolbox
SPM: Statistical Parametric Mapping
WM: White matter
GM: Gray matter
DPABI: Data Processing & Analysis for Brain Imaging
MNI: Montreal Neurological Institute
FD: Frame-wise displacement
AAL: Automated anatomical labeling
ROI: Regions of interest
NBS: Network-based statistic
SVM: Support vector machine
LOOCV: Leave-one-out cross-validation
ROC: Receiver operating characteristic curve
PHG: Parahippocampal gyrus
DCG.L: Left median cingulate and paracingulate gyri
SFGdor.R: Right dorsolateral superior frontal gyrus
SFGmed.L: Left medial superior frontal gyrus
TPOsup.R: Right temporal pole-superior temporal gyrus
TPOsup.L: Left temporal pole-superior temporal gyrus
IFGoperc.R: Right inferior frontal gyrus-opercular part
DTI: Diffusion tensor imaging
ASL: Arterial spin labeling
rCBF: Regional cerebral blood flow
FDG-PET: Fluorodeoxyglucose-PET
DELCODE: DZNE-Longitudinal Cognitive Impairment and Dementia
