# Supplementary material for: The compensatory phenomenon of the functional connectome related to pathological biomarkers in individuals with subjective cognitive decline
Source: Transl Neurodegener. 2020 May 27;9:21. doi: 10.1186/s40035-020-00201-6 (PMC7254770; doi:10.1186/s40035-020-00201-6)
Supplement: Supplementary file 1 — Additional file 1: Supplemental Fig. 1. Altered connections illustrated by rich club, feeder and local connections based on different thresholds. A. the top 10% node degree as threshold; B. the top 20% node degree as threshold. Abbreviations: SCD, subjective cognitive decline; HC, healthy control. * indicates a statistical difference between groups, p < 0.05. [file 40035_2020_201_MOESM1_ESM.docx]

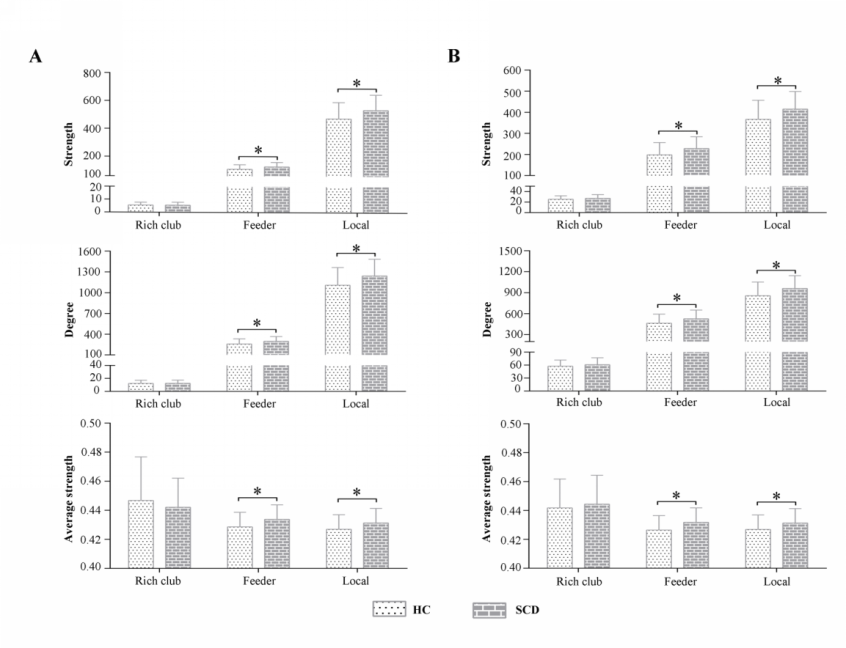
Supplemental Fig. 1. Altered connections illustrated by rich club, feeder and local connections based on different thresholds.
