# Supplementary material for: The compensatory phenomenon of the functional connectome related to pathological biomarkers in individuals with subjective cognitive decline
Source: Transl Neurodegener. 2020 May 27;9:21. doi: 10.1186/s40035-020-00201-6 (PMC7254770; doi:10.1186/s40035-020-00201-6)

Supplemental Fig. 2. The altered nodal shortest path length and nodal clustering coefficient between SCD and HC.


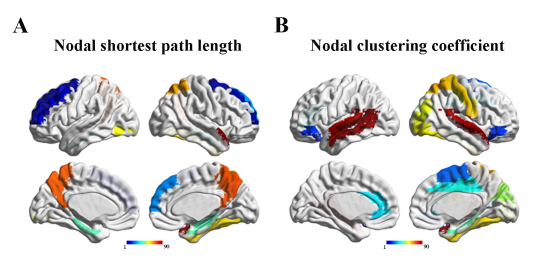

Supplement: Supplementary file 2 — Additional file 2: Supplemental Fig. 2. The altered nodal shortest path length and nodal clustering coefficient between SCD and HC. A. The SCD group showing significantly decreased nodal shortest path length in thirteen brain regions; B. The SCD group showing significantly increased nodal clustering coefficient in sixteen brain regions; Abbreviations: SCD, subjective cognitive decline; HC, healthy control; The color bar represents the label of brain regions in AAL-90 atlas. [file 40035_2020_201_MOESM2_ESM.docx]
