# Supplementary material for: The compensatory phenomenon of the functional connectome related to pathological biomarkers in individuals with subjective cognitive decline
Source: Transl Neurodegener. 2020 May 27;9:21. doi: 10.1186/s40035-020-00201-6 (PMC7254770; doi:10.1186/s40035-020-00201-6)
Supplement: Supplementary file 3 — Additional file 3: Supplemental Fig. 3. Relationships between altered network metrics and biomarkers in the HC group. No significance was found between the CSF Aβ1–42 and the nodal strength of PHG.L (r = 0.197, P = 0.345) (A), nodal global efficiency of the TPOsup.R (r = 0.069, P = 0.744) (B) and nodal local efficiency of the IFGoperc.R (r = − 0.046, P = 0.826) (C) in the HC group. Abbreviations: HC, healthy control; PHG.L, left parahippocampal gyrus; TPOsup.R, right temporal pole-superior temporal gyrus; IFGoperc.R, right inferior frontal gyrus-opercular part; CSF, cerebrospinal fluid; Aβ, amyloid-β. [file 40035_2020_201_MOESM3_ESM.docx]

Supplemental Fig. 3. Relationships between altered network metrics and biomarkers in the HC group.

**
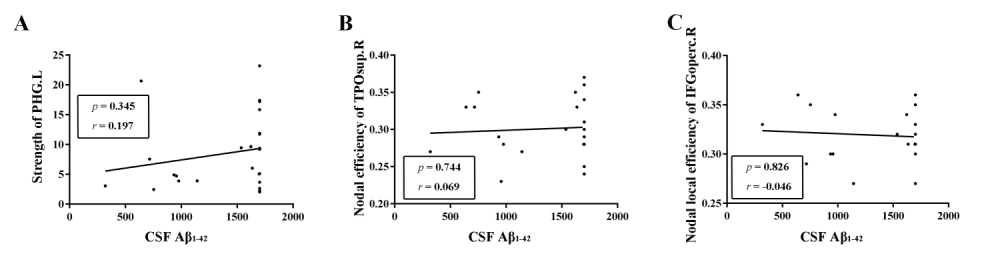
**
