# Supplementary material for: The compensatory phenomenon of the functional connectome related to pathological biomarkers in individuals with subjective cognitive decline
Source: Transl Neurodegener. 2020 May 27;9:21. doi: 10.1186/s40035-020-00201-6 (PMC7254770; doi:10.1186/s40035-020-00201-6)
Supplement: Supplementary file 5 — Additional file 5: Supplemental Table 2. The comparison of nodal properties between HC and SCD. The SCD group showed significantly increased nodal strength in the right superior frontal gyrus and the bilateral medial temporal lobe (P < 0.05, FDR corrected). The increased nodal global efficiency and nodal local efficiency mainly in the frontal, temporal and parietal regions were found in the SCD group (P < 0.05, FDR corrected). Abbreviations: SCD, subjective cognitive decline; HC, healthy control. [file 40035_2020_201_MOESM5_ESM.docx]

**Supplemental Table 2.**

| **The comparison of nodal properties between HC and SCD** | | | | |
| --- | --- | --- | --- | --- |
|  |  |  |  |  |
| **Nodal properties** | | **HC** | **SCD** | ***p value*** |
| **Nodal strength** | ORBsupmed.R | 11.62±5.72 | 15.64±7.05 | <0.001 |
|  | HIP.R | 8.70±5.71 | 12.77±7.51 | <0.001 |
|  | PHG.L | 9.79±6.39 | 14.92±7.66 | <0.001 |
|  | PHG.R | 10.60±6.47 | 15.76±7.85 | <0.001 |
| **Nodal global efficiency** | SFGdor.L | 0.30±0.04 | 0.32±0.03 | 0.002 |
|  | SFGdor.R | 0.31±0.04 | 0.33±0.03 | <0.001 |
|  | ORBsup.L | 0.29±0.04 | 0.31±0.05 | 0.010 |
|  | ORBsup.R | 0.30±0.04 | 0.32±0.04 | 0.008 |
|  | MFG.L | 0.31±0.04 | 0.33±0.03 | 0.001 |
|  | MFG.R | 0.31±0.03 | 0.33±0.03 | 0.007 |
|  | OLF.L | 0.25±0.07 | 0.29±0.06 | 0.008 |
|  | SFGmed.L | 0.29±0.04 | 0.31±0.04 | 0.004 |
|  | SFGmed.R | 0.29±0.04 | 0.31±0.04 | 0.003 |
|  | ORBsupmed.L | 0.28±0.05 | 0.31±0.05 | 0.002 |
|  | ORBsupmed.R | 0.29±0.04 | 0.31±0.05 | 0.001 |
|  | ACG.L | 0.30±0.04 | 0.32±0.04 | 0.007 |
|  | ACG.R | 0.31±0.04 | 0.32±0.04 | 0.013 |
|  | HIP.R | 0.26±0.05 | 0.29±0.06 | <0.001 |
|  | PHG.L | 0.27±0.05 | 0.31±0.05 | <0.001 |
|  | PHG.R | 0.28±0.05 | 0.32±0.05 | <0.001 |
|  | AMYG.L | 0.25±0.05 | 0.28±0.07 | 0.012 |
|  | MOG.L | 0.30±0.04 | 0.32±0.03 | 0.012 |
|  | IOG.L | 0.30±0.04 | 0.32±0.03 | 0.005 |
|  | FFG.L | 0.31±0.03 | 0.33±0.03 | 0.007 |
|  | FFG.R | 0.31±0.04 | 0.32±0.03 | 0.009 |
|  | SPG.L | 0.31±0.03 | 0.32±0.03 | 0.007 |
|  | SPG.R | 0.30±0.03 | 0.32±0.03 | 0.006 |
|  | PCUN.L | 0.32±0.02 | 0.33±0.02 | <0.001 |
|  | PCUN.R | 0.32±0.02 | 0.33±0.02 | 0.006 |
|  | PCL.L | 0.30±0.04 | 0.32±0.04 | 0.007 |
|  | TPOsup.L | 0.31±0.04 | 0.33±0.04 | 0.013 |
|  | TPOsup.R | 0.30±0.05 | 0.33±0.04 | 0.004 |
| **Nodal local efficiency** | MFG.R | 0.32±0.03 | 0.33±0.02 | 0.006 |
|  | IFGoperc.L | 0.32±0.03 | 0.34±0.02 | 0.005 |
|  | IFGoperc.R | 0.32±0.03 | 0.33±0.02 | 0.003 |
|  | IFGtriang.R | 0.31±0.04 | 0.33±0.03 | 0.005 |
|  | ORBinf.R | 0.31±0.04 | 0.32±0.02 | 0.002 |
|  | SMA.R | 0.31±0.03 | 0.32±0.02 | 0.014 |
|  | ACG.L | 0.31±0.04 | 0.33±0.03 | 0.006 |
|  | DCG.L | 0.30±0.03 | 0.32±0.02 | 0.009 |
|  | DCG.R | 0.30±0.03 | 0.32±0.02 | 0.003 |
|  | PHG.R | 0.30±0.05 | 0.33±0.04 | 0.002 |
|  | CUN.R | 0.32±0.02 | 0.33±0.03 | 0.012 |
|  | IOG.R | 0.32±0.04 | 0.33±0.03 | 0.009 |
|  | FFG.R | 0.31±0.03 | 0.33±0.02 | 0.011 |
|  | PoCG.R | 0.32±0.03 | 0.34±0.03 | 0.002 |
|  | SPG.R | 0.32±0.04 | 0.33±0.02 | 0.003 |
|  | IPL.L | 0.32±0.03 | 0.33±0.02 | 0.011 |
|  | IPL.R | 0.32±0.04 | 0.34±0.02 | 0.007 |
|  | SMG.L | 0.33±0.03 | 0.34±0.02 | 0.012 |
|  | SMG.R | 0.32±0.04 | 0.34±0.03 | 0.009 |
|  | ANG.R | 0.32±0.03 | 0.34±0.02 | <0.001 |
|  | PUT.R | 0.32±0.04 | 0.33±0.02 | 0.005 |
|  | PAL.R | 0.32±0.06 | 0.34±0.02 | 0.008 |
|  | STG.L | 0.31±0.03 | 0.32±0.02 | 0.002 |
|  | STG.R | 0.31±0.03 | 0.32±0.02 | 0.010 |
|  | TPOsup.R | 0.31±0.05 | 0.33±0.02 | 0.006 |
|  | MTG.L | 0.31±0.02 | 0.32±0.02 | 0.010 |
|  | MTG.R | 0.31±0.02 | 0.32±0.02 | 0.003 |
| Abbreviation: HC, health control; SCD, subjective cognitive decline. | | | | |
