# Supplementary material for: The compensatory phenomenon of the functional connectome related to pathological biomarkers in individuals with subjective cognitive decline
Source: Transl Neurodegener. 2020 May 27;9:21. doi: 10.1186/s40035-020-00201-6 (PMC7254770; doi:10.1186/s40035-020-00201-6)
Supplement: Supplementary file 6 — Additional file 6: Supplemental Table 3. The comparison of nodal shortest path length and nodal clustering coefficient between HC and SCD. The results of the nodal shortest path length and nodal clustering coefficient were described in this table. Abbreviations: SCD, subjective cognitive decline; HC, healthy control. [file 40035_2020_201_MOESM6_ESM.docx]

**Supplemental Table 3**

| **The comparison of nodal shortest path length and nodal clustering coefficient between HC and SCD** | | | | |
| --- | --- | --- | --- | --- |
|  |  |  |  |  |
| **Nodal properties** | | **HC** | **SCD** | ***p value*** |
| **Nodal shortest path length** | SFGdor.L | 3.39±0.60 | 3.12±0.38 | 0.003 |
|  | SFGdor.R | 3.33±0.54 | 3.06±0.32 | 0.001 |
|  | MFG.L | 3.31±0.49 | 3.07±0.33 | 0.002 |
|  | SFGmed.R | 3.49±0.56 | 3.24±0.44 | 0.005 |
|  | HIP.R | 4.01±0.84 | 3.59±0.79 | 0.003 |
|  | PHG.L | 3.82±0.68 | 3.36±0.69 | <0.001 |
|  | PHG.R | 3.74±0.68 | 3.27±0.64 | <0.001 |
|  | IOG.L | 3.40±0.42 | 3.21±0.36 | 0.005 |
|  | FFG.R | 3.32±0.41 | 3.14±0.29 | 0.006 |
|  | SPG.R | 3.34±0.40 | 3.17±0.26 | 0.005 |
|  | PCUN.L | 3.16±0.22 | 3.04±0.20 | 0.001 |
|  | PCUN.R | 3.17±0.23 | 3.06±0.20 | 0.005 |
|  | TPOsup.R | 3.37±0.56 | 3.12±0.48 | 0.006 |
| **Nodal clustering coefficient** | ORBinf.L | 0.31±0.06 | 0.34±0.05 | 0.007 |
|  | ORBinf.R | 0.32±0.06 | 0.35±0.05 | 0.006 |
|  | SMA.R | 0.33±0.07 | 0.36±0.06 | 0.004 |
|  | ACG.L | 0.32±0.07 | 0.35±0.05 | 0.008 |
|  | DCG.R | 0.31±0.06 | 0.34±0.07 | 0.003 |
|  | PHG.R | 0.32±0.07 | 0.35±0.07 | 0.003 |
|  | CUN.R | 0.34±0.05 | 0.37±0.07 | 0.007 |
|  | MOG.R | 0.34±0.06 | 0.37±0.06 | 0.008 |
|  | IOG.R | 0.34±0.07 | 0.37±0.06 | 0.004 |
|  | FFG.R | 0.32±0.06 | 0.36±0.05 | 0.002 |
|  | PoCG.R | 0.34±0.08 | 0.39±0.08 | 0.005 |
|  | SPG.R | 0.34±0.07 | 0.38±0.07 | 0.004 |
|  | STG.L | 0.32±0.06 | 0.36±0.06 | 0.001 |
|  | STG.R | 0.33±0.06 | 0.36±0.06 | 0.001 |
|  | TPOsup.R | 0.31±0.07 | 0.34±0.06 | 0.005 |
|  | MTG.L | 0.31±0.06 | 0.34±0.06 | 0.002 |
| Abbreviation: HC, health control; SCD, subjective cognitive decline. | | | | |
