# Supplementary material for: The compensatory phenomenon of the functional connectome related to pathological biomarkers in individuals with subjective cognitive decline
Source: Transl Neurodegener. 2020 May 27;9:21. doi: 10.1186/s40035-020-00201-6 (PMC7254770; doi:10.1186/s40035-020-00201-6)
Supplement: Supplementary file 7 — Additional file 7: Supplemental Table 4. The subnetwork derived from NBS analysis. A single connected subnetwork with 30 nodes and 35 connections exhibited higher connection strength in the SCD group than in the HC group (p < 0.001, corrected). Abbreviations: SCD, subjective cognitive decline; HC, healthy control. [file 40035_2020_201_MOESM7_ESM.docx]

**Supplemental Table 4.**

| **The subnetwork derived from NBS analysis** | | | | |
| --- | --- | --- | --- | --- |
|  |  |  |  |  |
| **Connection** | | **HC (mean value)** | **SCD (mean value)** | ***p value*** |
| **Region A** | **Region B** |  |  |  |
| PHG.L | PreCG.L | 0.08 | 0.22 | 8.52E-05 |
| PHG.R | PreCG.L | 0.06 | 0.19 | 3.36E-04 |
| AMYG.L | PreCG.L | 0.03 | 0.15 | 8.04E-05 |
| PHG.L | PreCG.R | 0.07 | 0.19 | 2.77E-04 |
| PHG.R | SFGdor.L | 0.05 | 0.22 | 2.07E-06 |
| ROL.R | ORBsup.L | 0.04 | 0.14 | 7.20E-04 |
| PCG.R | ORBsup.L | 0.09 | 0.22 | 4.11E-04 |
| PHG.L | ORBsup.L | 0.05 | 0.16 | 2.92E-04 |
| PHG.R | ORBsup.L | 0.03 | 0.13 | 1.34E-04 |
| SMG.R | ORBsup.L | 0.05 | 0.17 | 3.20E-04 |
| INS.L | MFG.L | 0.12 | 0.24 | 8.94E-04 |
| PHG.L | MFG.L | 0.05 | 0.16 | 4.96E-04 |
| PHG.R | MFG.L | 0.02 | 0.18 | 5.02E-07 |
| PHG.L | ORBmid.L | 0.03 | 0.13 | 4.50E-04 |
| PHG.L | IFGtriang.L | 0.04 | 0.16 | 1.51E-04 |
| PHG.R | IFGtriang.L | 0.04 | 0.15 | 3.45E-04 |
| ORBsupmed.R | ROL.R | 0.04 | 0.14 | 7.38E-04 |
| PHG.L | SMA.L | 0.06 | 0.17 | 9.36E-04 |
| THA.R | SMA.L | 0.12 | 0.24 | 7.79E-04 |
| PHG.R | SFGmed.L | 0.06 | 0.19 | 4.56E-05 |
| PUT.R | SFGmed.L | 0.06 | 0.20 | 1.87E-05 |
| PHG.R | SFGmed.R | 0.05 | 0.17 | 2.82E-04 |
| FFG.L | ORBsupmed.R | 0.11 | 0.23 | 7.78E-04 |
| PHG.L | DCG.L | 0.07 | 0.20 | 2.62E-04 |
| PHG.R | DCG.L | 0.06 | 0.20 | 1.33E-04 |
| PHG.L | DCG.R | 0.08 | 0.22 | 6.51E-05 |
| IOG.R | DCG.R | 0.11 | 0.24 | 8.68E-04 |
| CAL.L | PHG.L | 0.12 | 0.25 | 4.64E-04 |
| SOG.R | PHG.L | 0.07 | 0.19 | 4.91E-04 |
| SPG.R | PHG.L | 0.07 | 0.18 | 8.15E-04 |
| IPL.L | PHG.L | 0.06 | 0.17 | 5.15E-04 |
| PoCG.R | PHG.R | 0.07 | 0.19 | 9.69E-04 |
| IPL.L | PHG.R | 0.06 | 0.17 | 4.98E-04 |
| IOG.R | AMYG.L | 0.04 | 0.16 | 6.27E-05 |
| PCL.R | AMYG.L | 0.02 | 0.11 | 4.60E-04 |
| Abbreviation: HC, health control; SCD, subjective cognitive decline. | | | | |
