# Supplementary material for: The compensatory phenomenon of the functional connectome related to pathological biomarkers in individuals with subjective cognitive decline
Source: Transl Neurodegener. 2020 May 27;9:21. doi: 10.1186/s40035-020-00201-6 (PMC7254770; doi:10.1186/s40035-020-00201-6)
Supplement: Supplementary file 8 — Additional file 8: Supplemental Table 5. The features selected by SVM for HC VS SCD classification. Nodal properties and connections were utilized to classify whether a sample belonged to the SCD group. Abbreviations: SCD, subjective cognitive decline; HC, healthy control; SVM, support vector machine. [file 40035_2020_201_MOESM8_ESM.docx]

**Supplemental Table 5**

| **The features selected by SVM for HC VS SCD classification** | | | | | |
| --- | --- | --- | --- | --- | --- |
|  |  |  |  |  |  |
| **Feature** | | | **HC (mean value)** | **SCD (mean value)** | ***p* value** |
| **Nodal properties** | **Nodal strength** | | | | |
|  | ORBsupmed.R | | 11.62 | 15.64 | 5.12E-04 |
|  | HIP.R | | 8.70 | 12.77 | 7.14E-04 |
|  | PHG.L | | 9.79 | 14.92 | 6.28E-05 |
|  | PHG.R | | 10.60 | 15.76 | 7.90E-05 |
|  | **Nodal global efficiency** | | | | |
|  | SFGdor.R | | 0.31 | 0.33 | 4.78E-04 |
|  | HIP.R | | 0.26 | 0.29 | 5.43E-04 |
|  | PHG.L | | 0.27 | 0.31 | 5.76E-05 |
|  | PHG.R | | 0.28 | 0.32 | 1.89E-05 |
|  | PCUN.L | | 0.32 | 0.33 | 8.23E-04 |
|  | **Nodal local efficiency** | | | | |
|  | ANG.R | | 0.32 | 0.34 | 4.77E-04 |
| **Functional connections** | **Region A** | **Region B** |  | | |
|  | ORBsup.L | ROL.R | 0.04 | 0.14 | 7.20E-04 |
|  | ROL.R | ORBsupmed.R | 0.04 | 0.14 | 7.38E-04 |
|  | MFG.L | INS.L | 0.12 | 0.24 | 8.94E-04 |
|  | ORBsup.L | PCG.R | 0.09 | 0.22 | 4.11E-04 |
|  | PreCG.L | PHG.L | 0.08 | 0.22 | 8.52E-05 |
|  | PreCG.R | PHG.L | 0.07 | 0.19 | 2.77E-04 |
|  | ORBsup.L | PHG.L | 0.05 | 0.16 | 2.92E-04 |
|  | MFG.L | PHG.L | 0.05 | 0.16 | 4.96E-04 |
|  | ORBmid.L | PHG.L | 0.03 | 0.13 | 4.50E-04 |
|  | IFGtriang.L | PHG.L | 0.04 | 0.16 | 1.51E-04 |
|  | SMA.L | PHG.L | 0.06 | 0.17 | 9.36E-04 |
|  | DCG.L | PHG.L | 0.07 | 0.20 | 2.62E-04 |
|  | DCG.R | PHG.L | 0.08 | 0.22 | 6.51E-05 |
|  | PreCG.L | PHG.R | 0.06 | 0.19 | 3.36E-04 |
|  | SFGdor.L | PHG.R | 0.05 | 0.22 | 2.07E-06 |
|  | ORBsup.L | PHG.R | 0.03 | 0.13 | 1.34E-04 |
|  | MFG.L | PHG.R | 0.02 | 0.18 | 5.02E-07 |
|  | IFGtriang.L | PHG.R | 0.04 | 0.15 | 3.45E-04 |
|  | SFGmed.L | PHG.R | 0.06 | 0.19 | 4.56E-05 |
|  | SFGmed.R | PHG.R | 0.05 | 0.17 | 2.82E-04 |
|  | DCG.L | PHG.R | 0.06 | 0.20 | 1.33E-04 |
|  | PreCG.L | AMYG.L | 0.03 | 0.15 | 8.04E-05 |
|  | PHG.L | CAL.L | 0.12 | 0.25 | 4.64E-04 |
|  | PHG.L | SOG.R | 0.07 | 0.19 | 4.91E-04 |
|  | DCG.R | IOG.R | 0.11 | 0.24 | 8.68E-04 |
|  | AMYG.L | IOG.R | 0.04 | 0.16 | 6.27E-05 |
|  | ORBsupmed.R | PoCG.L | 0.11 | 0.23 | 7.78E-04 |
|  | PHG.R | PoCG.R | 0.07 | 0.19 | 9.69E-04 |
|  | PHG.L | SPG.R | 0.06 | 0.17 | 8.15E-04 |
|  | PHG.L | IPL.L | 0.06 | 0.17 | 5.15E-04 |
|  | PHG.R | IPL.L | 0.06 | 0.17 | 4.98E-04 |
|  | ORBsup.L | SMG.R | 0.05 | 0.17 | 3.20E-04 |
|  | ACG.L | PCUN.L | 0.11 | 0.23 | 5.75E-04 |
|  | HIP.R | PCUN.L | 0.09 | 0.24 | 4.03E-05 |
|  | HIP.R | PCUN.R | 0.08 | 0.21 | 1.27E-04 |
|  | AMYG.L | PCL.R | 0.02 | 0.11 | 4.60E-04 |
|  | SFGmed.L | PUT.R | 0.06 | 0.20 | 1.87E-05 |
|  | SMA.L | THA.R | 0.12 | 0.24 | 7.79E-04 |
|  | SFGdor.R | TPOsup.R | 0.11 | 0.25 | 1.14E-04 |
|  | ROL.L | ITG.R | 0.10 | 0.24 | 2.07E-04 |
| Abbreviation: HC, health control; SCD, subjective cognitive decline; SVM, support vector machine. | | | | | |
