# Supplementary material for: The compensatory phenomenon of the functional connectome related to pathological biomarkers in individuals with subjective cognitive decline
Source: Transl Neurodegener. 2020 May 27;9:21. doi: 10.1186/s40035-020-00201-6 (PMC7254770; doi:10.1186/s40035-020-00201-6)
Supplement: Supplementary file 9 — Additional file 9: Supplementary materials legends. Details regarding methods and materials. [file 40035_2020_201_MOESM9_ESM.doc]

**Supplementary material**

***Apolipoprotein E Genotyping***

Apolipoprotein E (APOE) genotypes of participants in this study were obtained from the ADNI database (http://adni.loni.usc.edu). The two SNPs (rs429358, rs7412) that define the epsilon 2, 3, and 4 alleles were genotyped using DNA extracted by Cogenics from a 3 ml aliquot of ethylenediaminetetraacetic acid (EDTA) blood. Polymerase chain reaction amplification was followed by HhaI restriction enzyme digestion, resolution on 4% Metaphor Gel, and visualization by ethidium bromide staining. All participants were classified as APOE +/+ (ε4/ε4), APOE +/- (ε4/ε2 and ε4/ε3) and APOE -/- (ε2/ε2, ε2/ε3 and ε3/ε3).

***Cerebrospinal fluid biomarkers***

Lumbar puncture and cerebrospinal fluid (CSF) sample preparation were performed as described in the ADNI manual (http://adni.loni.usc.edu/research/protocols/biospecimens-protocols/). Aβ_1-42_, t-tau, and p-tau were measured in each of the 416 CSF ADNI baseline aliquots using the multiplex xMAP Luminex platform (Luminex Corp, Austin, TX) with Innogenetics (INNO-BIA AlzBio3; Ghent, Belgium; for research use-only reagents) immunoassay kit-based reagents. Full details of this combination of immunoassay reagents and analytical platform are provided elsewhere. In brief, Innogenetics kit reagents included well-characterized capture monoclonal antibodies specific for Aβ_1-42_ (4D7A3), t-tau (AT120), and p-tau (AT270), each chemically bonded to unique sets of color-coded beads, and analyte-specific detector antibodies (HT7, 3D6). Calibration curves were produced for each biomarker using aqueous buffered solutions that contained the combination of three biomarkers at concentrations. Before performing these analyses of the ADNI and the independent autopsy-based CSF samples in the ADNI University of Pennsylvania ADNI Biomarker Core laboratory, an interlaboratory study was conducted to qualify the performance conditions, including all major variables that can affect the test results, for the immunoassay reagents and analytical platform.

***[^18^F] AV45 positron emission tomography scans***

[^18^F] AV45 positron emission tomography (PET) data were processed as described in the standardized protocol (http://adni.loni.usc.edu/methods/). Raw PET images from all sites are downloaded for quality control at the University of Michigan. Raw images are converted to a standard file format. Separate frames are extracted from the image file for registration purposes. Each extracted frame is co-registered to the first extracted frame of the raw image file. The base frame image and the five co-registered frames are recombined into a co-registered dynamic image set. These image sets have the same image size and voxel dimensions and remain in the same spatial orientation as the original PET image data. Each subjects co-registered averaged image from their baseline PET scan is then reoriented into a standard 160×160×96 voxel image grid, having 1.5 mm cubic voxels. These images are the result of smoothing of the above-mentioned images. Each image set is filtered with a scanner-specific filter function (can be a non-isotropic filter) to produce images of a uniform isotropic resolution of 8 mm FWHM, the approximate resolution of the lowest resolution scanners used in ADNI. Image sets from higher resolution scanners have been smoothed more than image sets from lower resolution scanners. The specific filter functions were determined from the Hoffman phantom PET scans that were acquired during the certification process. Mean florbetapir standard uptake value ratios (SUVRs) were computed within these brain regions (lateral and medial anterior frontal, lateral temporal, posterior cingulate, and lateral parietal cortex) and normalized to the whole cerebellum as the reference region.

***Network topological analyses***

The topological properties of the brain functional networks were analyzed using graph theory. We characterized the global network properties by the following parameters: network strength, clustering coefficient, shortest path length, small-worldness, global efficiency and local efficiency. In addition, we used nodal strength, nodal clustering coefficient, nodal shortest path length, nodal global efficiency and nodal local efficiency to describe the regional properties of the functional network.

***Global properties***

The network strength is the mean of the network strengths across all the nodes in the network, which can be defined as:

where N is the number of nodes in the network G, and S(i) is is the sum of the edge weights for the node i.

The clustering coefficient (Cp) of the network is the average clustering coefficient over all N nodes, which can be defined as:

$$Cp\left(G\right)=\frac{1}{N}\sum_{i\in G} C(i)$$

where N is the number of nodes in the network G, and C(i) is defined as the number of edges between its direct neighbors (denoted by subnetwork G_i_) divided by the total number of all possible edges.

The shortest path length (Lp) reflects the optimal path of information transfer from node i to node j, and quantifies the ability of parallel information propagation, which can be defined as:

where d_ij_ is the shortest path length between node i and j in the network G.

We scaled Cp and Lp derived from the networks to the mean Cp^rand^ and Lp^rand^ of 1000 random networks (i.e., γ = Cp/Cp^rand^ and λ = Lp/Lp^rand^) that preserved the same numbers of edges and nodes and degree distributions as the real networks to calculate the small-world properties (i.e., σ = γ/λ, which is typically >1 for small-world networks).

Global efficiency measures the parallel information transfer ability of the network, which can be defined as:

where N is the number of nodes in the network G, and d_ij_ is the shortest path length between node i and j in the network G.

Local efficiency measures the information exchanging ability among a subnetwork G_i_ with locally interconnected nodes and reflects system redundancy and tolerance to attack, which can be defined as:

where N is the number of nodes in the network G, and G_i_ is the subnetwork consisting of node i and its local neighbors.

In a hierarchical network, nodes with a low degree exhibit a higher clustering coefficient than nodes with a higher degree, reflecting a top-down organization. The hierarchy coefficient can be defined as:

*C*~ k^-β^

The hierarchy coefficient β can be estimated by fitting a linear regression line to the plot of log (C) versus log (k). The positive hierarchy coefficient β indicates a network in which nodes with high total connectivity have low local connectivity. The idea of hierarchy is closely linked to the concept of modularity.

The assortativity coefﬁcient is a correlation coefficient between the degrees of all nodes on two opposite ends of a link.

$$r=\frac{M^{-1}\sum_{i} j_{i}k_{i}-[M^{-1}\sum_{i} \frac{1}{2}(j_{i}+k_{i})]^{2}}{M^{-1}\sum_{i} \frac{1}{2}(j_{i}^{2}+k_{i}^{2})-[M^{-1}\sum_{i} \frac{1}{2}(j_{i}+k_{i})]^{2}}$$

where j_i_, k_i_ are the degrees of the vertices at the ends of the edge i, with i = 1,..., M.

Networks with a positive assortativity coefﬁcient are therefore likely to have a comparatively resilient core of mutually interconnected high-degree hubs. On the other hand, networks with a negative assortativity coefﬁcient are likely to have widely distributed and consequently vulnerable high-degree hubs.

***Nodal properties***

The nodal strength is defined as the sum of the edge weights in a subnetwork G_i_, which is the graph that includes the nodes that are direct neighbors of node i. The nodal strength evaluates the extent to which the node is connected to the remaining nodes in a network, which can be defined as:

where w_ij_ is the edge weight linking node i and j in the subnetwork G_i_.

The nodal clustering coefficient is the number of edges between its direct neighbors (denoted by subnetwork G_i_) divided by the total number of all possible edges, which can be defined as:

where N_i_ is the number of nodes in a subnetwork G_i_, and k_i_ is the the number of edges in the subnetwork G_i_.

The nodal shortest path length is the average of its shortest path lengths to all other nodes, which can be defined as

where d_ij_ is the shortest path length between node i and j in the network G.

The nodal global efficiency measures the average shortest path length between a given node i and all of the other nodes in the network, which can be defined as:

where N is the number of nodes in the network G, and d_ij_ is the shortest path length between node i and j in the network.

The nodal local efficiency measures the global efficiency of the subnetwork formed by this given node’s neighbors, which can be defined as:

where N_i_ is the number of nodes in a subnetwork G_i_, and d_mn_ is the shortest path length between node m and n in the subnetwork G_i_.

***Hub distribution***

In this study, we choose the top 15% (90 × 15% ≈ 14 ) brain regions with the highest nodal degree across all participants as rich club regions according to most of the previous studies. In addition, some researches also choose the top 10% or 20% node degree as threshold to confirm the stability of our results. Finally, we get the similar results (Supplemental Figure 1). Significant differences in the strength, degree and average strength of the feeder and local connections were identified, while no significant differences were found in rich club connections. In detail, the SCD group exhibited higher connections than the HC group (strength: feeder *p* = 0.002; local *p* = 0.002; degree: feeder *p* = 0.002; local *p* = 0.003; average strength feeder *p* = 0.037; local *p* = 0.048) based on the top 10% node degree as threshold (Supplemental Figure 1A). The SCD group showed higher connections than the HC group (strength: feeder *p* = 0.003; local *p* = 0.002; degree: feeder *p* = 0.004; local *p* = 0.002; average strength feeder *p* = 0.036; local *p* = 0.042) based on the top 20% node degree as threshold (Supplemental Figure 1B).

***Alterations in nodal shortest path length and nodal clustering coefficient***

The SCD group exhibited significantly decreased nodal shortest path length in thirteen brain regions (Supplemental Figure 2A) and increased nodal clustering coefficient in sixteen brain regions (Supplemental Figure 2B) (*P* < 0.05, FDR corrected, Supplemental Table 3).
